# Relationship(s) between obesity and inflammatory bowel diseases: possible intertwined pathogenic mechanisms

**DOI:** 10.1007/s12328-019-01037-y

**Published:** 2019-08-26

**Authors:** Andrew Szilagyi

**Affiliations:** Division of Gastroenterology, Department of Medicine, Jewish General Hospital, McGill University Medical School, 3755 Cote St Catherine Rd, Room E110, Montreal, QC H3T 1E2 Canada

**Keywords:** Obesity, Inflammatory bowel diseases, Clinical, Pathogenic relationships

## Abstract

The inflammatory bowel diseases, Crohn’s and ulcerative colitis have increased in incidence and prevalence from the mid-eighteen to the late nineteen centuries. From then to the current twenty-first century there has been a more rapid expansion of these disease to areas previously experiencing low rates. This latter expansion coincides with the current obesity pandemic which also began toward the end of the last century. Although the two diseases have radically different frequencies, there are interesting links between them. Four areas link the diseases. On an epidemiological level, IBD tends to follow a north–south gradient raising the importance of vitamin D in protection. Obesity has very weak relationship with latitude, but both diseases follow adult lactase distributions colliding in this plane. Is it possible that obesity (a low vitamin D condition with questionable response to supplements) reduces effects in IBD? On a pathogenic level, pro-inflammatory processes mark both IBD and obesity. The similarity raises the question of whether obesity could facilitate the development of IBD. Features of the metabolic syndrome occur in both, with or without obesity in IBD. The fourth interaction between the two diseases is the apparent effect of obesity on the course of IBD. There are suggestions that obesity may reduce the efficacy of biologic agents. Yet there is some suggestion also that obesity may reduce the need for hospitalization and surgery. The apparent co-expansion of both obesity and IBD suggests similar environmental changes may be involved in the promotion of both.

## Introduction

Inflammatory bowel diseases (IBD: comprising Crohn’s disease {CD} and idiopathic ulcerative colitis {UC}) are enigmatic inflammatory conditions of the gastrointestinal tract and associated extraintestinal manifestations. While the current pathogenic hypothesis includes host genetic predisposition, a dysregulated immune response in conjunction with a disordered intestinal microbiome, the specific interactive causes have not been precisely worked out. Multiple environmental variables are considered to modify risks for IBD [1, 2]. These diseases were rare at the beginning of the twentieth century. However, toward the end of the last century and continuing into the new millennium, IBD is increasing in areas of the world previously free of these diseases and may be peaking in incidence in western countries where the diseases originated [3].

Toward the end of the twentieth century, increasing body weight and obesity of populations progressed to epidemic proportions and the projected levels are predicted to increase in the next few decades [4]. Obesity is associated with numerous conditions including the metabolic syndrome (type 2 diabetes, ischemic vascular diseases, non-alcoholic fatty liver diseases: (NAFLD), hypertension and increased serum lipids) [5–9] and other diseases. These are listed in Table 1. Features of obesity which can promote other diseases include metabolic abnormalities due to the excess intake of energy and reduced utilization. These changes and diet (or other factors) have an important impact on the intestinal flora. Changes in the intestinal flora have taken a center stage in mediating multiple widely different diseases.

**Table 1:** Complications and diseases associated with obesity based on references [5–9]

Non-gastrointestinal complications	Gastrointestinal complications
Coronary heart disease [6, 7]	Gallstones [8]
High blood pressure [5–7]	Reflux esophagitis [9]
Stroke [5–7]	Barrett’s esophagus [9]
Type 2 diabetes [5–7]	Esophageal cancer [9]
Sleep apnea [5]	Nonalcoholic fatty liver [5–7]
Osteoarthritis [5]	Nonalcoholic steatohepatitis [5–7]
Infertility or irregular periods [5]	Colorectal cancer [9]
Kidney, prostate cancer [9]	Pancreatic cancer [9]
Uterine, ovarian, cervical, breast cancer [9]	Dyslipidemia [5–7]
Psychosocial disorders [5]	

The last 3–4 decades have seen a closer relationship emerging between IBD and the much more common condition of obesity. These changes encompass subtle geographic distributions of the former, an expansion into areas where IBD was previously scarce and possibly impacting on clinical features of IBD. These overlapping features suggest that environmental factors promoting both diseases have some common sources. This review will explore relationships and pathogenic features which can overlap between both.

## Epidemiologic overlap between IBD and obesity

The emergence of IBD is linked with the development of the industrial revolution in western countries [10]. Ulcerative colitis preceded Crohn’s disease. Since IBD has low mortality for extended periods of life, the current prevalence in the USA is around 0.5%. Toward the close of the twentieth century, IBD rates have increased significantly and may have plateaued in western countries but are increasing in emerging industrialized nations that have adopted western lifestyles. This includes Asia, Africa, and South America, although rates are still lower than in the west. Multiple factors related to genetics, diet with altered eating patterns and food processing, other environmental changes, smoking and increased hygienic practices are hypothesized to have contributed to increasing rates of IBD [3, 10].

The current pandemic of obesity appears to have started around the 1980s. Although some genetic predisposition increases risk, the main cause is thought to be related to changes in eating patterns (more processed foods increased reliance on prepared foods) and reduced physical activity [4]. In the last 3 decades, obesity as a primary problem of mainly the United States has gone global and is projected to involve a 1/4 of the existing world population by 2030 [11]. Obesity is the result of the intake of excess energy with reduced expenditure, leading to excess body fat. It may be defined in various ways, including fat mass or volume, abdominal circumference, waist to hip ratio in cm and most simply as body mass index (BMI) [12]. This latter term is based on body weight in kg divided by the height in meters squared (kg/m^2^). The world health organization defines overweight as BMI ≥ 25 kg/m^2^ and obese as ≥ 30 kg/m^2^ [12, 13]. In Asians where insulin resistance and complications can occur at lower body mass, the definition of obesity is defined at a lower value of ≥ 27.5 kg/m^2^ [14]. Although definition by BMI may not completely reflect body distribution of fat, and hence inaccurately predict complications of obesity, it seems the most appropriate to use in comparisons of population values. There are also gender differences in weight but these will not be stressed here. In addition, epidemiologic patterns apply to both overweight and obesity but only the latter is mentioned for the purposes of this review.

On a global, national level, IBD rates are linked with more education and social status. Generally, countries with increased gross domestic products (GDP; the total value of goods produced and services provided in a country during 1 year [15]) have higher IBD rates. On the other hand, rural attributes including growing up on a farm, exposure to farm animals and pets are linked with lower rates of disease. Expanding industrialization in developing nations is accompanied by rising rates of IBD. Thus, population density, such as that observed in urban centers, is also linked with increased risks for IBD [16, 17]. Such distributions are also evident in the expansion of IBD in former low-risk areas such as Asia [18].

The relationship of obesity with urban/rural distributions has both similarities and differences from IBD. There appears to be a bimodal relationship between income and obesity such that early on higher income is associated with the conditions. Later, poverty is more likely linked with obesity [19]. Depending from which country studies were carried out, early on, obesity is more associated with rural origins. This was noted in the United States [4, 20]. However, in recently developing countries, urban centers are more often linked with obesity [4, 21, 22], and are also linked with poverty [19, 23, 24].

### Geographic patterns compared between IBD and obesity

In the last 2 decades toward the end of the previous century, it was observed that many diseases in western countries had greater incidence at high as opposed to low latitudes.

Latitudes, in general, are stable geographic markers. In 1980, Garland and Garland published a seminal article on the possible inverse relationship between colorectal cancer mortality and exposure to sunshine [25]. Since sunshine is closely and inversely correlated with latitude, other diseases were examined for the relationship with latitudinal distributions. Subsequently, multiple diseases in industrialized nations were linked with a higher north–south (south–north in the southern hemisphere) gradient. The diseases included cancers [26, 27] hematological malignancies [28, 29] and autoimmune diseases like multiple sclerosis [30] and IBD [31, 32]. In the southern hemisphere, western type of diseases was initially observed mainly in Caucasians.

### Latitude, sunshine, vitamin D and diseases

Initially, the effect of latitude on disease rates was attributed to lack of sunshine (which correlates inversely to latitude) and its impact on vitamin D. Sunshine increases skin synthesis of vitamin D and low levels of this vitamin were indeed found to be related to increased incidence of many diseases. In vivo and in vitro studies supported the role of vitamin D. The discovery that vitamin D receptors (VDR) could be identified in many cells other than the skeleton reinforced the conclusion that this vitamin had other far-reaching effects on autoimmunity and cancer [33, 34]. Four VDR polymorphisms have been described which influence risks for IBD (TaqI, BsmI, FokI and ApaI) in different ways. These receptors have different effects on risks. For example, genotype ff of FokI increased the risk for UC in Asians, while ApaI, allele “a” protects against CD. Alternatively, the AA genotype of ApaI, the B allele of BsmI and the tt genotype of TaqI increase CD risks. These effects were noted to be different in populations from Asia and Europe. Another example is the protective effect of the T allele of TaqI against CD and UC in Europeans [35, 36].

However, in general, vitamin D has effects on the innate and adaptive immune system inhibiting proinflammatory Th1 cell and promoting anti-inflammatory Th2 cell development [37]. Normal functions of vitamin D also include appropriate function of nucleotide-binding oligomerizing domain protein 2 (NOD2) which expresses a number of antibacterial peptides protecting bacterial translocations [38]. Furthermore, vitamin D is involved in the expression of tight junction proteins which reduce intestinal permeability [39].

On clinical grounds, low serum vitamin D levels were associated with increased mortality [40]. However, interventional as opposed to observational studies have fared less well in showing a clear benefit of vitamin D supplementation. In a recent meta-analysis of 83 randomized controlled trials (RCT) of vitamin D in non-skeletal diseases, overall and cancer-related mortality were found to be lower. In addition, vitamin D supplements reduced rates of upper respiratory infections and asthma, but no impact was found in reducing the risk of a wide array of diseases [41].

In IBD low levels of vitamin D reduce adequate response to tumor necrosis α therapy [42]. Clinical outcome is modestly affected. A meta-analysis of 18 RCTs on vitamin D supplementation suggested that addition of the vitamin improved serum levels in a dose–response fashion. Seven of the 18 studies dealt with clinical remission rates. There was a significantly lower rate of relapse with active D supplement than placebo. These were noted in different studies at 3 and 12 months but not at 6 months. However, the dose of supplement did not impact on relapse rates and there were no significant increases in adverse effects. There were no reports on mucosal healing outcome [43].

### Latitude sunshine, vitamin D and obesity

When obesity is examined in relation with latitude there is an apparent global lack of association [44–46]. Hossain et al. plotted diabetes frequency by global regions (which closely follow the prevalence of obesity) based on data from 2000. Projections for 2030 suggest a possible relationship with lactase non-persistence (LNP) [11]. The relationship with lactase distributions is discussed further below. As a crude exercise, we used obesity frequencies reported by the Organization for Economic Co-operation and Development (OECD) from the years 2012 [47] and 2015 [48]. These data are based on both self-reported and measured values. Using Pearson correlations, national frequencies were evaluated for associations with national calculated latitudes, and annual sunshine exposure obtained from Ref. [49]. We also correlated obesity and reported LNP frequencies based on data from Storhaug [44] and some from Itan [45] and Szilagyi [49] (Table 2). It is noted that in both sets of obesity data there is a weak relationship with latitude or sunshine but a modest correlation with LNP. If the position taken is that a north/south gradient in the case of IBD (and other diseases) led to benefit of vitamin D, then the lack of association of obesity would suggest that vitamin D is less important in obesity.

**Table 2:** Pearson correlation coefficients among national frequencies of average calculated latitudes, yearly sunshine exposure rates [UVBKj/Y] [46], and national lactase non-persistence LNP rates based on references [44–46] with national frequencies of obesity obtained online for the years 2012 (OBn) and 2015 (OBm) from Obesity Update sites (OECD) ([47, 48], respectively)

Frequency obesity	Latitude	Sunshine (UVBKj/Y)	LNP rate
OBn2012	0.26 (*p* = 0.12) *N* = 36	− 0.06 (*p* = 0.8) *N* = 31	− **0.37** **(*p* = 0.02)** *N* = 37
OBm2015	0.16 (*p* = 0.4) *N* = 31	0.08 (*p* = 0.7) *N* = 30	**-0.34** **(*p* = 0.05)***N* = 32

The data on obesity contains both self-reported and measured values. Calculations are based on a range of data from available countries [*N*]. Correlation between the two sample years for the national frequency of obesity was 0.95

Although serum vitamin D level of less than 50 nmol/L is associated with obesity, there are mixed outcomes of studies evaluating the role of vitamin D [50]. The reasons for this may be due to the effects of vitamin D being different in pre-adipocytes compared with mature adipocytes and to variability due to differences in species of animals studied. The arguments and observations are reviewed by Dix et al. [51]. In small rodents and their in vitro cell models, vitamin D is reported to inhibit adipogenesis. However, in preadipocyte cultures from mice, vitamin D increases adipogenesis. There are few studies in humans and cell models but studies suggest increased adipocyte differentiation and accumulation of lipids [51]. The effects of vitamin D also work in conjunction with calcium. Intracellular calcium promotes increased lipid by enhanced lipogenesis in conjunction with vitamin D and as well a decrease in lipolysis.

To date, human studies in randomized controlled trials did not show that supplemental vitamin D decreased measures of adiposity when calcium intake was controlled and no calorie restrictions were imposed [52]. Review of in vitro and clinical trials led Dix et al. to conclude that there is insufficient evidence to state whether low vitamin D is due to volume distribution or whether it contributes to obesity or if observed effects are due to interactions with calcium [51].

### Other possible effects of latitude

The role of the intestinal microflora, the microbiome, has taken a center stage as a co-pathogenic mechanism in many diseases and is discussed later [53–57]. Both temperature and latitude itself have been suggested to alter the microbiome. A study from Norway suggested that summer temperatures decreased the development of ulcerative colitis by altering the microbiome [58]. In addition, industrialization has been linked with loss of microbial diversity (one of the components of microbial disarray, dysbiosis). The hypothesis has been put forth that latitude per se may be instrumental in this change [59]. The second relevant recent report is that a rapid loss of microbial diversity and function occurs among immigrants from non-western regions to the United States [60], that is immigrants take on the regional type of microbiome which is different from their native microbiome. Taken together, the effects of latitude on diseases may have several plausible explanations in addition to the effects of vitamin D.

### Lactase phenotypes

Another geographic marker which correlates with different western lifestyle diseases is the national distributions of adult lactase digestion ability. About 7–10 thousand years ago, evolutionary pressures, perhaps in response to cattle herding, allowed the emergence of the dominant genetic forms of lactase persistence. As such the adult population is divided into 1/3 dominant lactase-persistent people (LP able to digest lactose into adulthood) and 2/3 recessive lactase non-persistent (LNP loses ability to digest lactose after a variable time in childhood or early adulthood) [61]. There are distinct geographic LP/LNP distributions in the populations of the world and these also correlate with different diseases.

In 1986, Nanji and Denardi published a hypothesis where they suggested LNP status may protect against IBD. Based on data from 12 countries available from 1960 to 1970 at that time, they showed a statistically significant correlation of − 0.93 for CD and − 0.89 for UC with increasing LNP proportions [62]. Several publications by our group reexamined relations between national LNP frequency distributions and a number of other diseases which also correlated with latitude [49, 63–65]. Among these, IBD was included. Crohn’s disease and ulcerative colitis incidence and prevalence rates were obtained for 55 countries (with some missing data). Approximate average national latitudes and yearly national sunshine (ultraviolet B, UVB) exposure were calculated. Although accuracy was somewhat compromised particularly for large countries, these reports confirmed correlations of IBD with both geographic markers [63, 64] as shown in Table 3. Although such a comparison is not validated due to different methodologies and available national rates, the large difference from Nanji and Di Nardi [62] is suggestive of change from the earlier correlations. Nevertheless, CD and UC retained their relationship with latitude as well.

**Table 3:** Correlations of IBD rates with geographic markers

	UC	LNP	UVB	Latitude
CD	0.75	− 0.73	− 0.53	0.56
UC	1	− 0.59	− 0.38	0.44
LNP		1	0.74	− 0.76
UVB			1	− 0.98

Spearman correlation coefficients (*r* values) of Crohn’s disease incidence (CD), ulcerative colitis incidence (UC) both from approximately the year 2000 as a mean value source, average calculated national ultraviolet B exposure (UVB), average calculated national latitude and estimated national lactase non-persistence (LNP). All values were statistically significant at *p* < 0.05. Table is partly reproduced from Ref. [49]

### Other genetic associations with lactase phenotype, economics

The explanations for relationship of “western” type diseases with LP/LNP distributions is not obvious and may be spurious, but is consistent. Initially, it was hypothesized that co-evolution of other genes with lactase persistence may predispose to other diseases like IBD. The genes include the NOD2/CARD15 polymorphisms [66], the cystic fibrosis sodium transporter and the human leukocyte antigen system [67–69]. The relationship between ancient genes and predisposition to modern diseases has been previously hypothesized [70].

However, a number of findings contradict a protective effect of LNP for IBD. The rapid increase in rate and the effect of early age of onset of IBD in immigrants to high-risk areas [71, 72] tend to support changes in the microbiome which may be affected both by industrialization and latitude or temperature as described. Also a meta-analysis of lactose maldigestion observed in patients with IBD failed to show a bias toward LP participants [73].

Other observed correlations include national economics. The GINI index (named after its creator, C Gini) is a numerical ratio of inequality of wealth distribution in a country. The range is 0–1 where 0 is perfect distribution of wealth throughout the population of the country and 1 means that one person has all the national wealth [74]. In general, lower GINI indices suggest a fairer distribution of national wealth and are characteristic of western nations. Both Crohn’s disease and ulcerative colitis incidence are modestly correlated with the GINI index. In this exercise, the Pearson correlations were LNP vs. GINI, *r* = 0.67, *p* < 0.0001, CD vs. GINI, *r* = − 0.44, *p* = 0.0049; and UC vs. GINI, *r* = − 0.41, *p* = 0.0139 (data calculated but not published, based on refs [64, 74]). These correlations support the observation that better socioeconomic economic development promotes IBD [10, 17, 18].

The LP/LNP ratios are not fixed but depend on population migrations as can be seen in the most recent publications of national lactase distribution rates [44]. Therefore, the relationships between diseases and LNP can be explained by a number of factors and the specific relevance remains unclear.

## Inflammation induced by obesity which could abet IBD

### Metabolic effects

Obesity predisposes to various diseases (some outlined in Table 1) through interaction of several major pathogenic pathways. The metabolic aspects relate to interactions between the development of insulin resistance (at the muscle, liver and fat cells leading to increased pancreatic insulin secretion) driven largely by adipocyte produced hormones or “adipokines”. There is evidence that the pro-inflammatory cytokine TNFα inhibits tyrosine kinase activity in the insulin receptor which contributes to insulin resistance [75]. Adiponectin, the most common, produced largely by visceral fat cells has a dampening effect on cytokines like TNFα and IL-6 and is often decreased with increased fat mass. This allows an increase in the pro-inflammatory response and thus contributes to insulin resistance [76]. Leptin also a product of adipocytes and cells of the placenta also promotes inflammation and affects satiety [77, 78]. In obesity, there may be increased leptin resistance or rarely a genetic deficiency. Leptin and adiponectin are thought to have opposing effects on cancer and Alzheimer’s both of which are of increased risk with central obesity [79]. Another recently described adipokine, resistin is found in the reticuloendothelial system promoting insulin resistance and a proinflammatory response. It is thought to promote cardiovascular effects of obesity and diabetes [80]. Several other adipokines interact to promote or protect against insulin resistance [81, 82].

Development of hyperglycemia and insulin resistance induce oxidative stress leading to cellular damage. In turn, this promotes low-grade inflammation. Thaiss et al. described a mouse model in which hyperglycemia is the primary cause which alters the gut barrier through reprogramming transcription in intestinal epithelial cells. These changes lead to alterations of tight junction and adherence integrity. The vehicle for this effect was shown to be related to the GLUT2 glucose transporter. Barrier alterations lead to bacterial product and enteric infection translocations in the model and correction of hyperglycemia improves barrier permeability. In the case of humans, glycated hemoglobin correlates with systemic infections supporting a similar role of hyperglycemia in exerting effects on the gut barrier. The relationship with hyperglycemia was independent of obesity [83].

### Microbiome effects

Whether low-grade inflammatory response in the obese host promotes intestinal microbiome changes or bacterial alterations promote obesity is still unclear although lean/obese bacterial differences are discernable and fecal transplant from obese animal models can induce obesity [55, 84, 85]. Similarly, in IBD, the intestinal microbiome reflects or plays a pathogenic role in disease causation. Intestinal flora comprises bacteria, viruses, fungi and archaea and has complicated interactions which are only beginning to be investigated and recognized [86–89]. The intestinal microbiome functions as a major modulator for both the innate and adaptive immunity. Changes in bacterial composition also lead to changes in gut barrier function. A shift in the microbiome composition leading to dysbiosis in obesity as well as in IBD is the core of potential pathogenic impact on the host [90]. In this state, there is a reduction of diversity and richness of bacteria as well as changes in bacterial taxa with reduction of protective bacteria and the emergence of pathobionts (potential pathogenic bacteria). These taxonomic changes are being evaluated in different diseases. Although mucosal adherent microbiota may be more relevant, at this time most studies of the microbiome are derived from studies on stool. Table 4 outlines comparisons of several phyla, genera and species found in association with obesity and inflammatory bowel diseases, mostly derived from stool samples of patients [53, 91–106]. These are incomplete and there are some discrepancies among studies. Nevertheless, those listed are consistent and several phyla and genera show similar trends while others show opposite directions in change. Furthermore, specific species in the three conditions vary.

**Table 4:** Colonic microbial findings compared in obesity with IBD

Bacterial taxa	Obesity	Crohn’s disease	Ulcerative colitis
Firmicutes (P)	Increased [91]	Decreased [53, 91]	Decreased [53, 91]
Bacteroides (P)	Decreased [91]	Decreased [53, 91]	Increased [53] or Decreased [91]
Proteobacteria (P)	***Increased*** [95]	***Increased*** [53, 91]	***Increased*** [53, 91]
Actinobacter (P)	Decreased [91]	Increased [91]	Increased [91]
*Faecalibacterium prausnitzii* G + S	***Decreased*** [91]	***Decreased*** [53, 97]	***Decreased*** [53, 96]
Roseburia (G)	Increased [91, 101]	Decreased (inulinivorans) [97]	Decreased (hominis) [96]
*Clostridium (leptum)* G + S	Increased [105] or ***Decreased*** [106]	***Decreased*** [104]	***Decreased*** [104]
*Akkermansia muciniphila* G + S	Decreased [92, 99]	Decreased in patients <16 years Only [93]	Stable Compared with healthy controls [93]
Bifidobacteria G	***Decreased*** [91, 102]	Increased [93] **or*Decreased*** [94, 100]	Increased [93] or ***Decreased*** [94, 100]
*Escherichia coli* G + S	***Increased*** [102]	***Increased*** [53]	***Increased*** [98]
*Ruminococcus gnavus* G + S	***Increased*** [95]	***Increased*** [103]	***Increased*** [103]
Desulfovibrio G	Decreased [99]	Increased [53]	Increased [53]

Several bacterial taxa that have been reported comparing phyla (P), genus (G) and species (S) levels from intestinal microflora found in obesity and IBD (Crohn’s disease (CD), ulcerative colitis (UC)) are shown with accompanying references in brackets. The reports are based on bacteria derived from stool [91, 92, 94–97, 99–103, 105, 106] and some from mucosal biopsies [93, 98, 104]. Similarly reported findings of bacterial directions are marked in bold and italicized. A meta-analysis of dysbiotic taxa comparing obesity and IBD showed that IBD was more consistent than findings in obesity [91]

Major differences in bacterial phyla between obesity and IBD which seem consistent are the increase in Firmicutes while Actinobacter is decreased. In dysbiosis of both IBD and obesity, there is a shift in bacteria from obligate to facultative anaerobes. This observation is supported by the increases in *E. coli* (enterobacteriaceae) [53, 98] and *R. gnavus* [103] which are both facultative anaerobes. The changes putatively accompany increased oxidative stress. In addition, short-chain fatty acid producers (SCFA) such as *F. prausnitzii* [53, 96, 97] and Bifidobacteria [91, 94, 100, 102] are reduced as well, but reporting of such changes in IBD is somewhat variable [93]. Some of the variations in Bifidobacteria are at species level but these bacteria also have important effects on immunity.

A feature of obesity appears to be an increase in the SCFA producer Roseburia which could provide more energy to the host [91, 101]. However, Roseburia are decreased relative to controls in type 2 diabetes [106] and in animal models on high-fat diets [107]. They are also reduced in IBD [96, 97]. As such, there exists not only differences but also similarities in dysbiosis of obesity and IBD. In IBD, the microbial dysbiosis is more characteristic. Joosens et al. identified five characteristic bacterial changes in Crohn’s disease [108]. The pattern reported in CD included a decrease in *Dialister invisus*, *Bifidobacterium adolescentis*, *F. prausnitzii* and a species of Clostridium cluster XIV (not further specified here) and increased *R. gnavus*. In UC, while *F. prausnitzii* is found to be decreased, there is also a reduction of *Roseburia hominis* which is different from the dysbiosis of CD [96] where Roseburia (inulinivorans) is identified [97]. A meta-analysis of the studies of dysbiosis in obesity and IBD have found that bacterial signatures in IBD are more distinct than in obesity [91].

Although the pathogenic significance of dysbiosis is still not clear, there are studies to suggest that the observed changes in IBD exert pathogenic effects. A direct role of the dysbiotic microbiome found in IBD patients is supported by animal studies. When IBD patients’ microbiome is transplanted into humanized gnotobiotic mice some of the pro-inflammatory features characteristic of IBD is reproduced in these mice [109]. In another mouse model, a high-fat diet which normally induces obesity also reduced Paneth cell numbers and interfered with antimicrobial protein production and reduced Goblet cell number and mucin secretion. Intestinal permeability was also increased. Mice transplanted with feces from high-fat fed mice were more susceptible to dextran sulfate sodium [DSS]-induced colitis [110]. These studies support (but not prove) a direct pathogenic role of dysbiosis as primary rather than secondary in disease.

**Fig. 1:**
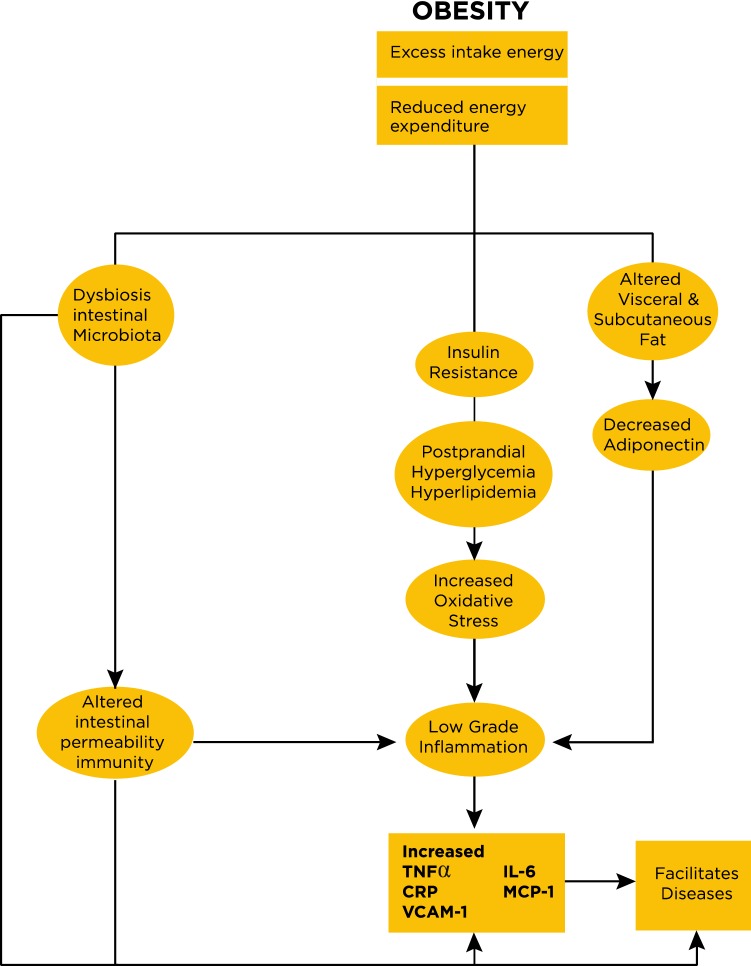
Pathogenic contributions associated with obesity are shown figure based on and modified from reference [46]. The primary drivers of host pro-inflammatory response originate from adipokines produced from visceral and subcutaneous fat. Reduced levels of adiponectin play a permissive role in the release of pro-inflammatory cytokines (*TNFα* tumor necrosis factor alpha, *CRP* C-reactive protein, IL-6 *interleukin-6*, vascular cell adhesion molecule-1-VCAM 1 and monocyte chemoattractant protein-1-MCP-1) [75–78, 80, 83]. Insulin resistance is promoted also by the interaction of TNFα and insulin receptor which decreases tyrosine kinase [75]. Insulin resistance promotes oxidative stress which also can impact on intestinal bacteria. In addition, alterations in the intestinal microbiome contribute to dysbiosis as well as to altered intestinal permeability which then, in turn, contributes to promoting the pro-inflammatory state [84, 85]. Low-grade inflammation facilitates diseases like inflammatory bowel diseases

Taken together these microbial changes demonstrate the effects of oxidative stress in both IBD and obesity raising the possibility that obesity facilitates IBD. It is, however, not clear whether these bacterial patterns are not a general phenomenon reflecting oxidative stress in other diseases as well where pathogenesis is attributed to changes in the microbiome. Figure 1 outlines these processes.

## Comorbidities in obesity shared with inflammatory bowel disease

Several co-morbidities associated with obesity have become recognized to be associated with IBD also. An important association noted between IBD and obesity concerns fatty liver or NAFLD (non-alcoholic fatty liver disease). A recent assessment of the world population prevalence of NAFLD reported a range of 9–43%, with about 27% in the USA [111]. Among obese people, the risk of NAFLD was reported to be 3.5 times higher with a clear dose–effect of BMI, than in non-obese people [112].

In IBD, a combined prevalence of 23% of fatty liver was reported in a systematic review for UC based on a total of 13 studies. In the same review, CD, based on four studies, a statistically higher combined prevalence was reported for NAFLD; 39.5% compared with 20% (range 6–33%) in the normal population although the study did not provide BMI values for the controls [113]. The diagnosis of NAFLD was based on different modalities including ultrasound or biopsies during colectomy or necropsy or volunteers with normal liver tests were carried out. Thus, CD seems to predispose to NAFLD more than UC.

In a prospective study of 321 patients with CD followed for more than 3 years, 33.6% developed fatty liver but only 0.9% were considered obese at the beginning. This represented an incidence of 9.1/100 patient-years. A total of 7 (2.2%) patients developed advanced liver disease [114]. A more recent study reported a prevalence of 32.8% NAFLD with 12.2% with more advanced fibrosis diagnosed by transient elastography [115]. In this study, 30.4% of participants were overweight and 13.8% were obese (BMI ≥ 30 kg/m^2^). There were no comparative control population data.

Factors putatively predisposing to fatty liver in IBD were found to be age, a higher BMI, and higher serum triglycerides [115]. Although disease activity, duration and prior surgery were also reported to impact on NAFLD [114], other studies did not find a close relationship with these variables instead the presence of metabolic syndrome was more closely related to liver involvement [116, 117]. In the latter study, no significant differences were found between non-IBD and IBD patients when BMI was compared, but IBD patients had an 18.5% morbid abdominal circumference compared with 6.8% in patients without IBD [117]. While medications like corticosteroids can predispose to fatty liver, these latter studies failed to find an association of NAFLD with medications in IBD [116, 117].

Furthermore, NAFLD has been described in underweight patients both in non-IBD and IBD [118, 119]. The pathogenesis in non-obese and obese NAFLD patients, however, is similar. The type and distribution of visceral compared to subcutaneous fat may be related without involving BMI. The hallmark of fatty liver is insulin resistance in both, although, the specific mechanisms are still elusive. In both obesity and IBD, the release of TNFα may have an impact on insulin receptors [75]. In addition, several genetic polymorphisms contribute even without the presence of metabolic syndrome [118].

Also, the contribution of the intestinal microbiome with altered intestinal permeability and bacterial translocations has emerged as a major factor in pathogenesis of NAFLD. Detailed changes in the microbiome with advancing stages of NAFL to NASH cirrhosis have been recognized [120].

Other consequences of obesity and the metabolic syndrome have also been evaluated in IBD. The occurrence of cardiovascular events (ischemic strokes and myocardial infarcts) is reported as conflicting. An earlier meta-analysis found no increased mortality but did find increased cardiovascular disease in IBD patients [121], while another study found no effects [122]. Some studies found an overall inverse relationship between IBD and coronary artery disease [123, 124]. However, there was a trend for increased mesenteric ischemia and a trend for more frequent dysrhythmias in younger women in the latter study [124]. In the most recent meta-analysis evaluating > 2.6 × 10^5^ IBD patients and > 5.5 × 10^6^ healthy controls for cardiovascular risk, modest risk of ischemic heart disease was reported in both Crohn’s disease and ulcerative colitis. The risk was higher in younger women less than 50 years old. In sub-analyses, controlling for obesity or smoking did not influence the outcome [125]. In a smaller retrospective controlled cohort study spanning 30 years from Olmsted county, USA, the risk for both acute myocardial infarct (CD and UC) and heart failure (UC) was found to be increased. Use of corticosteroids more than doubled risk [126].

Type 2 diabetes which is a common co-morbid state with obesity has been evaluated both in Crohn’s disease and ulcerative colitis. A British study followed patients with different inflammatory conditions including CD and UC compared with matched healthy controls for period of 11 years and found a modest 26% increased risk for type 2 diabetes in UC [127]. Shared co-morbidities between obesity and IBD could result from triggering of immunity by energy imbalance and perpetuation by infiltration of visceral fat. In IBD, immunity is dependent on genetics and the environment mediated via the microbiome. Inflammation is perpetual and chronic in obesity and intermittent with flares in IBD. Adipokines appear to play a role in instigating inflammation in both obesity and IBD. The perpetuation of inflammation increases risks for co-morbid conditions shared by the two diseases [128]. These processes could link the epidemiology of these two conditions.

## Impact of obesity on clinical outcome of IBD

Despite a pro-inflammatory environment promoted by obesity, the clinical impact on IBD is reported to be variable. A study evaluating the relationship between obesity at initial diagnosis of IBD reported on 524 patients and found that Crohn’s patients were more likely to have higher BMI than UC patients. However, the relationship also showed that there was an inverse relationship with BMI and CD as opposed to UC. A dose–response was demonstrated with diagnosing CD and increasing frequency of obesity [129]. Crohn’s disease was more of a risk after the age of 45 in obese subjects.

In a prospective cohort study of more than 300,000 Europeans initially free of IBD the development of CD and UC was monitored between 1991 and 1998. Subsequently, over the follow-up period, 177 developed UC and 75 developed CD, but the BMI had no association with either UC or CD [130]. This study did not suggest a pathogenic link between the two conditions. However, the possibility needs further evaluation.

When examining the development of obesity-related co-morbidities the relationship between the two is not easily separated. As noted the prevalence of obesity in Crohn’s disease is reported to be less [114] or similar [115, 117, 131] to prevalence in the general population when assessing for NAFLD. However, the precise reason behind the relationship of NAFLD with IBD remains unclear [132]. The risk of ischemic heart disease in IBD seems to be independent of obesity, but does not follow risks necessarily as in non IBD patients [125].

One debated area is over the possible adverse effect of obesity on response to biological therapy. A reduction in clinical outcome and lower trough levels in response to the TNFα antibody medications for Infliximab [133] and Adalimumab [134, 135] have been reported. However, not all studies concurred with this observation [136]. A recent meta-analysis of therapeutic outcome with different biological agents in various inflammatory disorders reported that the risk of therapeutic failure was 60% higher in obese patients. However, this therapeutic failure was not statistically significant in IBD [137]. More recently trough levels of the α_4_β_7_ integrin blocking antibody vedolizumab were reported to be reduced by the presence of obesity as defined by BMI [138].

Similarly, the impact of obesity on the clinical outcome of IBD is also conflicting and was reviewed previously [139]. In UC obesity increased risk of surgery and in CD, risk seemed to be less as reported in the study from Scotland [131]. In another study, obesity was found more often in UC and women. While markers of metabolic syndrome and C-reactive protein were more frequently increased, no association between BMI and need for hospitalization, surgery or medication alterations were noted [140]. Contrarily in CD, the presence of metabolic syndrome increased the risk of hospitalization twofold [141]. In addition a report from France suggested that obese patients with CD had more perianal disease and needed more hospitalizations [142], but in a further study a more mild disease course was found in obese patients with CD [143].

Most recently the topic was discussed in a systematic review. Seven studies were included evaluating obesity and IBD (5 studies) or CD (2 studies). Targets of interest were surgery need, perianal disease, use of more advanced medical therapy and need for hospitalization. The study concluded that IBD patients with obesity were significantly less likely to undergo IBD related surgery, receive hormonal therapy and need hospitalization than nonobese IBD patients. Other target variables showed no statistically significant differences [144]. This meta-analysis suggests that obesity possibly reduces severity of IBD or that sicker IBD patients are not obese.

The similarities in pathogenic mechanisms and the clinical overlap in comorbidities, support a hypothesis that obesity may promote IBD [120]. However, evidence is conflicting and confusing. It is somewhat counterintuitive that there would be a link between these two diseases. IBD is an energy-wasting disease while obesity enhances energy utilization with diminished expenditure. The ideal support of evidence would be determining that obesity would precede more cases of IBD than normal-weight individuals. Several studies evaluated overweight and obesity in young adults. The Nurse’s Health Study II found that obesity at the age of 18 increased risk for CD but not UC [145]. Another cross-sectional study found similar results [120], as did a Danish National Birth cohort study [146]. Two other studies examined the influence of BMI on IBD risk from Denmark. The first evaluated BMI in children from the age of 7–13 and found that there was a graded increase in risk of CD with increasing BMI, but only until the age of 30 [147]. There was an inverse relationship with UC. In a follow-up study on male Danish conscription examinations, the BMI at this time period had a “*U*”-shaped effect on risk of CD until the age of 60. However, only low BMI (< 18.5) was significantly associated. The inverse association with UC was also significant in this study [148]. Firm conclusions about IBD promotion by obesity requires more proof, but is suggestive.

## Summary and conclusions

Obesity is a world pandemic which started in the last part of the twentieth century and is increasing in prevalence [149]. The pro-inflammatory pathogenic mechanisms and dysbiotic intestinal microbiota associated with obesity are both harbingers and promoters of many non-communicable diseases. Among diseases, IBD began in the mid-nineteenth century along with the early phases of the industrial revolution. Both CD and UC, however, became more common and widespread around the time of the start of the obesity pandemic. Western industrialization and lifestyle appear to be the main forces behind IBD. While obesity has its roots in western society extension into less industrialized regions suggest other factors may also be at play.

It is somewhat counterintuitive that there would be a link between these two diseases. IBD is an energy-wasting disease while obesity enhances energy utilization with diminished expenditure. Yet the relationships between these two diseases are complex and can be divided into 4 planes.

Epidemiological features that appear both opposite (obesity more poverty, IBD higher social status,) but also similar (disease of high population densities at least in emerging industrialized areas). A glaring difference is the relationship of IBD with latitude and the lack of association in the case of obesity. However, both diseases retain a relationship with lactase distributions. On a hypothetical level, this observation raises the question of the lack of a role of vitamin D in obesity. It also raises a question regarding the effect of vitamin D on IBD when the two diseases are expanding in parallel directions.

The second important area of interaction relates to similar pro-inflammatory mechanisms noted both in IBD and obesity. As outlined, this raises the question whether obesity may produce mechanisms which could facilitate the acquisition of IBD.

The third interaction is the apparent development of similar co-morbid conditions (NAFLD, cardiovascular disorders and perhaps type 2 diabetes) in both. Hypothetically, the existence of similar complications reflect environmental factors which promote the metabolic syndrome in both obesity and IBD. Given the current importance of the microbiome and research, suspicion of pathogenesis points to this variable.

The fourth area of impact is the possibility that obesity may modify the clinical course of IBD. On one level, the therapeutic benefits of biological agents may be reduced by obesity. Alternatively outcome and some complications may be reduced. This latter observation, however, may be also explained by reverse causation where obese patients with IBD are less sick through increased nutrition? Further evaluation of the potential relationships between obesity and IBD will be of interest.
